# Tumor suppressor immune gene therapy to reverse immunotherapy resistance

**DOI:** 10.1038/s41417-021-00369-7

**Published:** 2021-08-05

**Authors:** Sunil Chada, Dora Wiederhold, Kerstin B. Menander, Beatha Sellman, Max Talbott, John J. Nemunaitis, Hyo Min Ahn, Bo-Kyeong Jung, Chae-Ok Yun, Robert E. Sobol

**Affiliations:** 1 MultiVir Inc, Houston, TX USA; 2 University of Toledo Medical Center, Eleanor N. Dana Cancer Center, Toledo, OH USA; 3 Hanyang University, Seoul, South Korea; 4 Institute of Nano Science and Technology (INST), Hanyang University, Seoul, South Korea

**Keywords:** Tumour immunology, Drug development, Tumour immunology

## Abstract

**Background:**

While immune checkpoint inhibitors are becoming a standard of care for multiple types of cancer, the majority of patients do not respond to this form of immunotherapy. New approaches are required to overcome resistance to immunotherapies.

**Methods:**

We investigated the effects of adenoviral p53 (Ad-p53) gene therapy in combination with immune checkpoint inhibitors and selective IL2 or IL15 CD122/132 agonists in the aggressive B16F10 tumor model resistant to immunotherapies. To assess potential mechanisms of action, pre- and post- Ad-p53 treatment biopsies were evaluated for changes in gene-expression profiles by Nanostring IO 360 assays.

**Results:**

The substantial synergy of “triplet” Ad-p53 + CD122/132 + anti-PD-1 therapy resulted in potential curative effects associated with the complete tumor remissions of both the primary and contralateral tumors. Interestingly, contralateral tumors, which were not injected with Ad-p53 showed robust abscopal effects resulting in statistically significant decreases in tumor size and increased survival (*p* < 0.001). None of the monotherapies or doublet treatments induced the complete tumor regressions. Ad-p53 treatment increased interferon, CD8^+^ T cell, immuno-proteosome antigen presentation, and tumor inflammation gene signatures. Ad-p53 treatment also decreased immune-suppressive TGF-beta, beta-catenin, macrophage, and endothelium gene signatures, which may contribute to enhanced immune checkpoint inhibitor (CPI) efficacy. Unexpectedly, a number of previously unidentified, strongly p53 downregulated genes associated with stromal pathways and IL10 expression identified novel anticancer therapeutic applications.

**Conclusions:**

These results imply the ability of Ad-p53 to induce efficacious local and systemic antitumor immune responses with the potential to reverse resistance to immune checkpoint inhibitor therapy when combined with CD122/132 agonists and immune checkpoint blockade. Our findings further imply that Ad-p53 has multiple complementary immune mechanisms of action, which support future clinical evaluation of triplet Ad-p53, CD122/132 agonist, and immune checkpoint inhibitor combination treatment.

## Background

Immune checkpoint inhibitor therapy has become a new standard of care for multiple recurrent and metastatic cancers. However, most cancer patients do not respond to this form of treatment [1]. Various approaches are being tested to increase immune checkpoint blockade efficacy, including a combination with immune-stimulating cytokines [2, 3]. Interleukin 2 (IL2) and interleukin 15 (IL15) belong to a family of immune-stimulating cytokines sharing a common beta chain (CD122) and gamma-chain (CD132) receptor known to drive the proliferation and cytolytic activity of CD8 + T cells and natural killer (NK) cells. Selective CD122/132 agonists have been developed with minimal alpha chain (CD25) binding that mitigates the generation of capillary leak toxicities and the induction of immune-suppressive T regs [4–7].

TP53 is the prototypic tumor suppressor that regulates responses to a wide range of cell stressors, including cell cycle arrest, cellular senescence, apoptosis, DNA damage repair, hypoxia, oncogenic stress, and epithelial-mesenchymal transition (EMT) [8]. Ad-p53 is a replication impaired adenoviral vector encoding expression of the wild-type p53 tumor suppressor protein, which has demonstrated antitumor effects in preclinical and clinical studies as a monotherapy and in combination with other treatment modalities [9–13].

We evaluated Ad-p53 tumor suppressor therapy in a murine tumor model known to be highly resistant to immunotherapy in combination with IL2/IL15 CD122/132 agonists and immune checkpoint blockade. We observed substantial synergy supporting further development of triplet Ad-p53, CD122/132 agonist, and immune checkpoint inhibitor combination treatment.

## Methods

### Animals, tumor inoculation, and measurements

C57BL/6 (B6) mice (6–8 weeks of age, ten animals per treatment group) were injected subcutaneously into the right flank with B16F10 melanoma cells (ATCC, 5 × 10^5^ cells/mouse) to form the “Primary Tumor.” Treatment started when tumors reached approximately 60 mm^3^ in size (designated Day 1). On day 14, animals were inoculated on the contralateral side with B16F10 cells to form “Secondary tumor,” and primary and secondary tumor growth followed for up to 60 days. Animals were sacrificed when tumors reached ~2000 mm^3^.

### Viral vectors

Replication-deficient human type 5 adenovirus (Ad5) encoding for expression of p53 tumor suppressor gene was used. The construction, properties, and purification of the vector have been reported elsewhere for Ad-p53 vectors [11]. Four doses of viral vectors or PBS were administered, intratumorally, at 48-h intervals (5 × 10^9^ viral particles/dose, in 50 µl total volume).

### Anti-PD-1 treatment

Animals were treated with intraperitoneal anti-PD-1(10 mg/kg) every 3 days starting on Day 1, over a 30-day period following implantation of the primary tumor. The anti-PD-1 monoclonal antibody clone RMP1-14 was obtained from Bioxcell, West Lebanon, NH. Outcome measures were subcutaneous tumor growth, measured twice per week by caliper measurements. The tumor growth was monitored by measuring the length (L) and width (w) of the tumor, and tumor volume was calculated using the following formula: volume = 0.523 L(w)^2^. Treatment outcomes were evaluated by measurement of tumor volumes in primary and contralateral tumors and their statistical analyses by *t* test, analysis of variance (ANOVA), Kruskal-Wallis ANOVA, and by comparisons of survival using Kaplan–Meier and log-rank tests.

### CD122/ CD132 agonist treatment

To generate the CD122/CD132 agonist (IL-2 based), recombinant murine IL-2 (eBioscience or R&D Systems Minneapolis, MN), and S4B6-1 antimouse IL-2 antibody (Bioxcell, West Lebanon, NH or BD Biosciences) were combined to form immunocomplexes before administration. The immunocomplexes were prepared by incubating the anti-IL-2 monoclonal with IL-2 for 15 min at room temperature. The murine IL-2 (eBioscience or R&D Systems Minneapolis, MN) was mixed with the S4B6-1 antimouse IL2 antibody (Bioxcell, West Lebanon, NH or BD Biosciences) at a molar ratio of 2:1. The IL-2/S4B6 mAb immunocomplexes were administered intraperitoneally (IP) at 2.5 µg IL-2/dose on days 2, 6, and 10 (palpable tumors were identified as day 0). Similarly, to generate the IL-15 based CD122/ CD132 agonist, immunocomplexes were prepared from recombinant murine IL-15 (eBioscience) and recombinant soluble murine IL-15Rα-Fc (R&D Systems). The immunocomplex was suspended in 0.1% bovine serum albumin (BSA)/PBS, mixed, and incubated for 30 min at 37 °C before injection. The reagent is 2 μg of IL-15 complexed with 12 μg of IL-15Rα-Fc in 300 μL 0.1% bovine serum albumin (BSA)/PBS and was similarly injected IP after tumors became palpable. These IL2 and IL15 immune cytokine complexes have selective CD122/132 receptor activation with reduced toxicities and greater efficacy than their native IL2 or IL15 cytokines [2, 3, 14–16].

### Nanostring transcriptome gene-expression analyses of Ad-p53 treatment

We also describe in this report, the initial transcriptome results of gene-expression profiles induced by Ad-p53 treatment performed as part of a new clinical trial combining Ad-p53 and anti-PD-1 or anti-PD-L1 in patients with recurrent head and neck squamous cell carcinoma (HNSCC) and other solid tumors approved for immune checkpoint inhibitor therapy NCT03544723. RNA was isolated from pre- and post-treatment samples and compared using Nanostring IO 360 gene-expression panel (Nanostring Technologies Seattle, WA). This panel tests expression of 770 genes involved in neoplasm pathology, tumor microenvironment, and cancer immune responses. Samples were processed and analyzed as described [17]. mRNA expression was measured with the nCounter technology, provided by NanoString Technologies. nCounter uses probes with barcodes attached to DNA oligonucleotides that directly bind to RNA. The sample preparation and analyses were performed according to the manufacturer’s protocol using The PanCancer IO 360 gene-expression panel that includes 770 genes. Gene-expression signatures were defined as described previously [17, 18]. The normalization was performed by correcting for the expression of technical controls and 30 housekeeping genes included in the panel. nCounter gene-expression data were obtained for pre- and post-treatment biopsies.

### Statistical analysis

Graph Pad Prism 8.0 software was employed for statistical analyses. A statistical analysis of variance (ANOVA) was employed to compare treatment effects on tumor size. For survival comparisons, Kaplan–Meier survival estimates, and the log-rank test were utilized. Fisher’s exact test was employed for the comparison of complete remission rates. The statistically significant *p* values were less than or equal to 0.05. All statistically significant results were confirmed in repeated experiments.

## Results

### Ad-p53 has local and abscopal effects with reversal of anti-PD-1 resistance

**Fig. 1:**
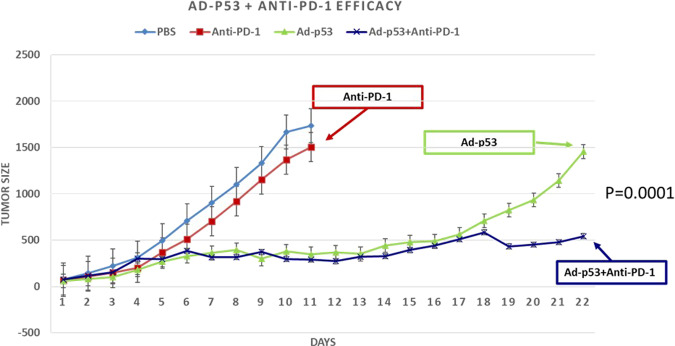
Local effects on Ad-p53-injected tumors + anti-PD-1 therapy. Ad-p53 + anti-PD-1 combination treatment-induced statistically significant decreases in tumor growth compared to either anti-PD-1 or Ad-p53 therapy alone. The evaluation of tumor growth using ANOVA statistical analysis confirmed synergistic effects of the combination treatment over either agent used as monotherapy (*p* = 0.0001). Anti-PD-1 monotherapy had minimal efficacy with substantial tumor progression similar to animals control PBS treatment. The treatment with Ad-p53 monotherapy resulted in significantly delayed tumor growth and Ad-p53 + anti-PD-1 combination therapy reversed anti-PD-1 treatment resistance. The tumor size = mm^3^ and was measured after treatments were started (designated Day 1) when tumors reached approximately 60 mm^3^ in size.

We evaluated the ability of Ad-p53 to reverse resistance to immune checkpoint inhibitor therapy in the B16F10 melanoma tumor model, which is known to be refractory to immunotherapy. Mimicking clinical applications, we allowed tumors to progress on anti-PD-1 treatment before intratumoral administration of Ad-p53 therapy to tumors in one flank with contralateral tumors not injected with Ad-p53. The local treatment effects on the Ad-p53-injected tumor are shown in Fig. 1. Ad-p53 + anti-PD-1 combination treatment-induced statistically significant decreases in tumor growth compared to either anti-PD-1 or Ad-p53 therapy alone. The evaluation of tumor growth using ANOVA statistical analysis confirmed synergistic effects of the combination treatment over either agent used as monotherapy (*p* = 0.0001). We observed only minimal anti-PD-1 monotherapy efficacy with results similar to the tumor progression seen for control PBS treatment. In contrast, treatment with Ad-p53 monotherapy resulted in significantly decreased tumor growth and Ad-p53 + anti-PD-1 combination therapy reversed anti-PD-1 treatment resistance (see Fig. 1). The treatment with a combination of Ad-luciferase (Ad-Luc) and anti-PD-1 did not enhance the effect of anti-PD-1 therapy (see Supplemental Fig. 1).

**Fig. 2:**
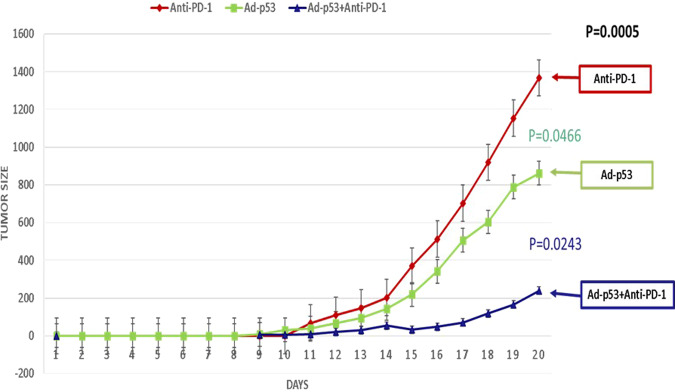
Abscopal effect of Ad-p53 in contralateral tumors not injected with Ad-p53. Contralateral tumor volume over time in mice whose primary tumor had received either anti-PD-1, Ad-p53, or a combination of Ad-p53 + anti-PD-1 treatment. Consistent with the synergistic effect observed in the suppression of the primary tumor growth, we also observed a statistically significant abscopal effect with decreased growth in the contralateral (secondary) tumors that did not receive tumor suppressor therapy. These findings imply that the combination treatment (Ad-p53 + anti-PD1) induced systemic immunity mediating the abscopal effects. Contralateral tumors in animals whose primary tumor had been treated with Ad-p53 alone showed significantly delayed tumor growth (*p* = 0.046) compared to the growth rate of the primary tumors treated with anti-PD-1 alone. An even greater abscopal effect on contralateral tumor growth (*p* = 0.0243) was observed in mice whose primary tumors were treated with combined Ad-p53 + anti-PD-1. Tumor size = mm^3^ and was measured from the day of tumor implantation.

As shown in Fig. 2, abscopal, systemic antitumor effects of localized Ad-p53 treatment were observed in contralateral tumors that were not injected with Ad-p53. Consistent with the synergistic effect seen in the suppression of Ad-p53 injected tumors, we also observed a statistically significant abscopal effect with decreased growth in the contralateral tumors that did not receive Ad-p53 tumor suppressor therapy. These findings imply that the combination treatment (Ad-p53 + anti-PD1) induced systemic immunity mediating the abscopal effects. Contralateral tumors in animals whose primary tumor had been treated with Ad-p53 alone showed significantly delayed tumor growth (*p* = 0.046) compared to the growth rate of the primary tumors treated with anti-PD-1 alone. An even greater abscopal effect on contralateral tumor growth (*p* = 0.0243) was observed in mice whose primary tumors were treated with combined Ad-p53 + anti-PD-1.

**Fig. 3:**
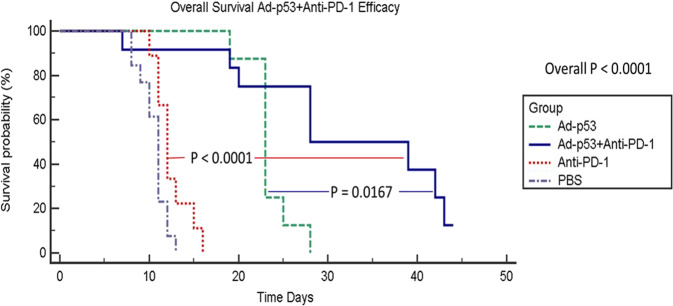
Kaplan–Meier survival curves showing superior efficacy for Ad-p53 + anti-PD-1 therapy. Kaplan–Meier survival curves for mice treated with either PBS, anti-PD-1, Ad-p53, or a combination of these agents. The results show no significant difference in the survival of animals treated with PBS or anti-PD-1, increased survival in those treated with Ad-p53, and a significant enhancement of survival in animals treated with a combination of Ad-p53 + anti-PD-1 over that observed in mice treated with either Ad-p53 (*p* = 0.0167), or anti-PD-1 (*p* < 0.001) monotherapy.

With respect to survival, combined Ad-p53 and anti-PD-1 therapy demonstrated a statistically significant increase in survival compared to Ad-p53 therapy alone (*p* = 0.0167) and anti-PD-1 therapy alone (*p* < 0.001) (see Fig. 3). Consistent with the synergistic effects on tumor growth, the increase in median survival for the combined Ad-p53 and anti-PD-1 group was more than additive compared to the effects of Ad-p53 and anti-PD-1 treatments.

### Local and systemic efficacy by “Triplet” Ad-p53 + immune checkpoint blockade + CD122/133 agonist therapy

**Fig. 4:**
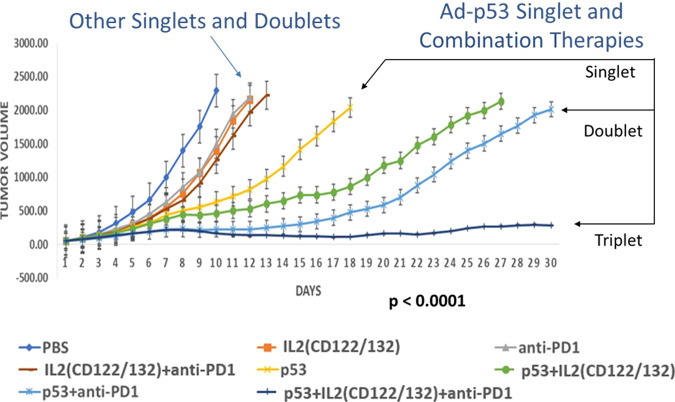
Substantially superior efficacy of “Triplet” Ad-p53 + IL2 CD122/132 agonist + anti-PD-1 therapy. There was significantly enhanced efficacy of Triplet Ad-p53 + CD122/132 + anti-PD-1 treatment compared to any of the other singlet or doublet therapies. Importantly, a statistical analysis of variance (ANOVA) comparison of tumor volumes on Day 30 determined that the synergy of the antitumor effects was only maintained in the “Triplet” Ad-p53 + CD122/132 + anti-PD-1 treatment combination (*p* value <0.0001 overall and *p* value <0.0001 separately compared to every other treatment group). Tumor size = mm^3^ and was measured after treatments were started (designated Day 1) when tumors reached ~60 mm^3^ in size.

**Fig. 5:**
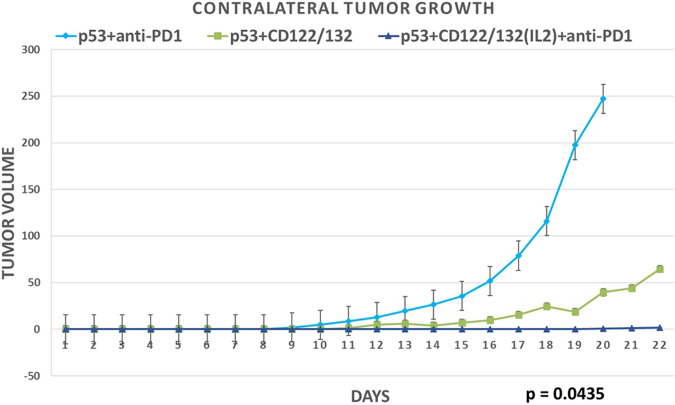
Substantially superior abscopal/systemic efficacy of “Triplet” Ad-p53 + IL2 CD122/132 agonist + anti-PD-1 therapy. Consistent with the unexpected, substantially increased synergistic effects of Ad-p53 + CD122/132 + anti-PD-1 treatment on the primary tumor growth, we also observed a surprisingly powerful and statistically significant abscopal effect of triplet Ad-p53 + CD122/132 + anti-PD-1 treatment compared to the other Ad-p53 treatment groups. A statistical analysis of variance (ANOVA) comparison of these contralateral tumor volumes determined synergy of the anti-tumor effects of Ad-p53 + CD122/132 + anti-PD-1 treatment (*p* value = 0.0435 overall). Only the Ad-p53 + CD122/132 + anti-PD-1 group demonstrated a statistically significant decrease in contralateral tumor growth vs. the Ad-p53 + anti-PD-1 group (*p* value = 0.0360). Tumor size = mm^3^ and was measured from the day of tumor implantation.

**Fig. 6:**
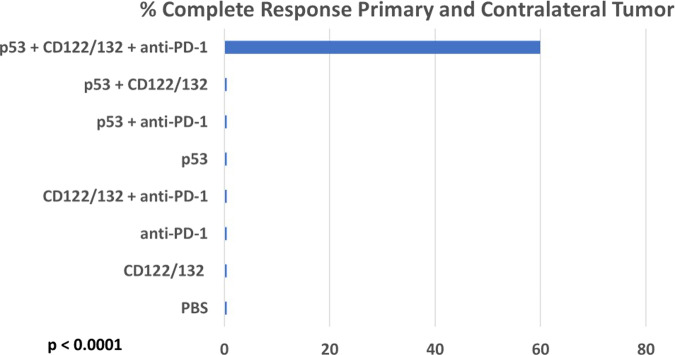
“Triplet” Ad-p53 + CD122/132 + anti-PD-1 treatment induces complete tumor responses. Only the Ad-p53 + CD122/132 + anti-PD-1 treatment resulted in complete tumor remissions of both the primary and contralateral tumors. The complete tumor responses of both the primary and contralateral tumors were observed in 60% of the Ad-p53 + CD122/132 + anti-PD-1 treatment group (six of ten animals) and there were no complete tumor responses in any of the 70 animals in the other treatment groups (*p* value <0.0001 by two-sided Fisher’s Exact test comparing Ad-p53 + CD122/132 + anti-PD-1 treatment group vs. animals in all other treatment groups; *p* value <0.011 by two-sided Fisher’s Exact test comparing Ad-p53 + CD122/132 + anti-PD-1 treatment group vs. any other treatment group).

In a subsequent series of experiments, we evaluated the effects of combining IL2 or IL15 CD122/CD132 agonists with Ad-p53 tumor suppressor and immune checkpoint blockade. The treatment efficacy was evaluated by assessing tumor volumes (in primary and contralateral tumors), the complete tumor response rates, and survival. The results demonstrated unexpected, substantial synergy of the “Triplet”Ad-p53 + CD122/132 + anti-PD-1 therapies that resulted in potentially curative treatment associated with the complete tumor remissions of both the primary and contralateral tumors with significantly superior abscopal effects on distant tumors not injected with Ad-p53 tumor suppressor therapy. As demonstrated in Figs. 4, 5, and 6, the triplet Ad-p53 + CD122/132 + anti-PD-1 therapy was the only treatment that resulted in complete tumor remissions and long-term survival.

### Significant improvement of local efficacy of “Triplet” Ad-p53 + immune checkpoint blockade + CD122/133 agonist therapy in the primary tumor injected with Ad-p53

In regard to the primary tumor volume as shown in Fig. 4, there was enhanced efficacy of Ad-p53 + CD122/132, Ad-p53 + anti-PD-1, and Ad-p53 + CD122/132 + anti-PD-1 treatments compared to any of the therapies alone. Importantly, a statistical analysis of variance (ANOVA) comparison of tumor volumes on Day 30 determined that the synergy of the antitumor effects was only maintained in the “Triplet” Ad-p53 + CD122/132 + anti-PD-1 treatment combination (*p* value <0.0001 overall and *p* value <0.0001 separately compared to every other treatment group). There was severe tumor progression during CD122/132, anti-PD-1, and CD122/132 + anti-PD-1 therapies, which were reversed by combination with Ad-p53 therapy. There was variation between the primary tumor growth rates for the PBS controls shown in Figs. 1 and 4. This is most likely the result of the highly exponential growth characteristics of the B16F10 tumor which can account for larger variations between experiments than for other tumor models.

### Superior abscopal/systemic effects of “Triplet” Ad-p53 tumor suppressor immune therapy

As shown in Fig. 5, abscopal, systemic antitumor effects in contralateral tumors that were not injected with Ad-p53 were significantly superior for Ad-p53 “Triplet” therapy. Figure 5 depicts contralateral tumor volumes over time in mice receiving the three most effective tumor treatments with either the combination of Ad-p53 + IL2 CD122/132, Ad-p53 + anti-PD-1, or Ad-p53 + IL2 CD122/132 + anti-PD-1. A statistical analysis of variance (ANOVA) comparison of these contralateral tumor volumes determined synergy of the antitumor effects of Ad-p53 + IL2 CD122/132 + anti-PD-1 treatment (*p* value = 0.0435). Similar results were observed with IL15 derived CD122/132 treatments (see Supplemental Fig. 2).

### “Triplet” Ad-p53 + CD122/132 + anti-PD-1 treatment induces complete tumor responses

It is generally appreciated that the complete tumor responses to therapy are associated with important therapeutic benefits and are required for curative outcomes. As shown in Fig. 6 for the p53 treatment groups and their controls, only Ad-p53 + CD122/132 + anti-PD-1 treatment resulted in the complete tumor remissions of both the primary and contralateral tumors. The complete tumor responses of both the primary and contralateral tumors were observed in 60% of the Ad-p53 + CD122/132 + anti-PD-1 treatment group (six of ten animals) and there were no complete tumor responses in any of the 70 animals in the other treatment groups (*p* value <0.0001 by two-sided Fisher’s Exact test comparing Ad-p53 + CD122/132 + anti-PD-1 treatment group vs. animals in all other treatment groups; *p* value <0.011 by two-sided Fisher’s Exact test comparing Ad-p53 + CD122/132 + anti-PD-1 treatment group vs. any other treatment group). Unexpectedly, the complete tumor responses were durable and were maintained after 40 days in 50% of the Ad-p53 + CD122/132 + anti-PD-1 treatment group presumably curing these animals of these tumors. Taken together, these findings indicate that of all the Ad-p53 therapies, only the triplet combination Ad-p53 + CD122/132 + anti-PD-1 treatment resulted in curative efficacy by inducing powerful local and systemic antitumor immunity mediating substantial abscopal effects.

### “Triplet” Ad-p53 + CD122/132 + anti-PD-1 treatment results in extended survival

**Fig. 7:**
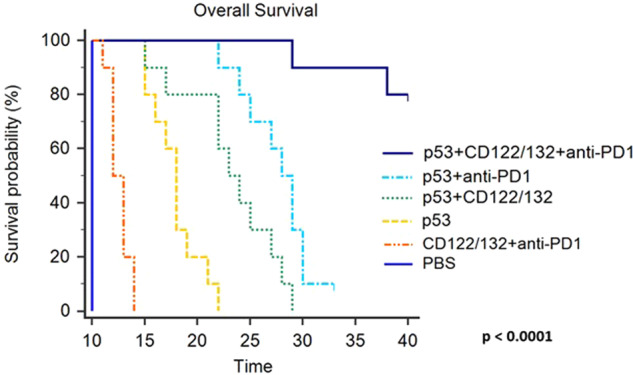
“Triplet” Ad-p53 + CD122/132 + anti-PD-1 treatment extends survival. Kaplan–Meier survival curves for mice treated with either PBS, CD122/132 + anti-PD-1, Ad-p53, or the combination of Ad-p53 + CD122/132, Ad-p53 + anti-PD-1 and Ad-p53 + CD122/132 + anti-PD-1. There was a statistically significant difference in these survival curves by the log-rank test (*p* < 0.0001 overall; *p* value <0.0003 comparing Ad-p53 + CD122/132 + anti-PD-1 treatment group vs. any other treatment group). These results also demonstrate an unexpected, substantial synergy of Ad-p53 + CD122/132 + anti-PD-1 therapy. The median survival of the Ad-p53 + CD122/132 + anti-PD-1 therapy group had not been reached after 40 days and 80% of this treatment group were still alive without evidence of any remaining tumors. In stark contrast, 98% (49/50) of animals in the other treatment groups had died by Day 30 and had median survivals ranging between 10 and 28 days.

The Kaplan–Meier survival curves shown in Fig. 7 demonstrate the substantial synergy of triplet Ad-p53 + CD122/132 + anti-PD-1 therapy compared to mice treated with either PBS or Ad-p53 monotherapy, or the doublet therapies CD122/132 + anti-PD-1, Ad-p53 + CD122/132 or Ad-p53 + anti-PD-1. There was a statistically significant difference in these survival curves by the log-rank test (*p* < 0.0001 overall; *p* value <0.0003 comparing Ad-p53 + CD122/132 + anti-PD-1 treatment group vs. any other treatment group). The median survival of the triplet Ad-p53 + CD122/132 + anti-PD-1 therapy group had not been reached after 40 days and 80% of this treatment group were still alive. In stark contrast, 98% (49/50) of animals in the other treatment groups had died by Day 30 and had median survivals ranging between 10 and 28 days.

In summary, these findings indicate that of all the Ad-p53 therapies, only the triplet combination Ad-p53 + CD122/132 + anti-PD-1 treatment resulted in potentially curative efficacy and long-term survival by inducing synergistic local and systemic antitumor immunity with substantial abscopal effects.

### Gene-expression profiles induced by Ad-p53 treatment

To assess the gene-expression profiles most modulated by Ad-p53 treatment, mRNA isolated from pre- and post-Ad-p53 treatment biopsies in a patient with recurrent HNSCC were compared using the Nanostring IO 360 gene-expression panel. The Nanostring IO 360 dataset was analyzed for genes substantially up- or downregulated as a result of p53 treatment defined by a greater than or less than tenfold change from baseline.

**Fig. 8:**
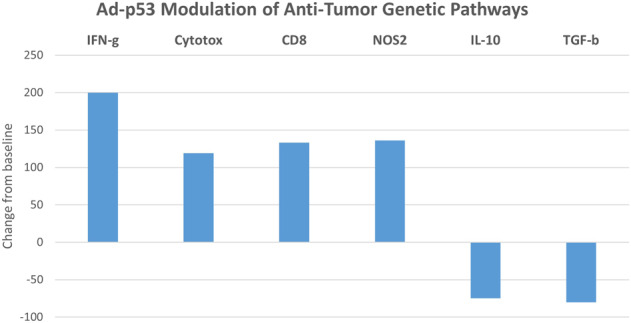
Concomitant upregulation of immune activating and downregulation of immune-suppressive/stromal gene pathways. Ad-p53 treatment increased immunstimulatory interferon-gamma (IFN-g), Cytotoxicity (Cytotox), CD8 + T cells, and nitric oxide synthase 2 (NOS2) signatures while decreasing expression of immune inhibitory and stroma forming interleukin 10 (IL-10) and transforming growth factor- beta (TGF-b) signatures.

A total of 23 strongly modulated genes out of the 770 gene set met these criteria. These genes with at least a tenfold change in expression represented a highly, statistically significant gene subset most substantially effected by p53 treatment (*p* value <0.00001 by two-sided Fisher’s Exact test compared to genes with less than a tenfold change from baseline). These genes may be grouped into immune modulatory, stroma/fibrosis, and tumor suppressor/cell cycle functional categories as listed in Table 1 and Fig. 8. Unexpectedly, many of these genes were found to be involved in immune responses and antistroma/fibrosis functions which are not typically associated with p53 tumor suppressor mechanisms of action.

**Table 1:** Strongly modulated genes effected by p53 treatment with at least a tenfold change in expression.

GENE	Fold regulation	Function	Immune activation	Antistroma	Tumor suppression cell cycle
**SOX2**	42.3	Transcription factor associated with superior HNSCC prognosis; HPV repression			+
**S100A8**	35.8	Calgranulin—inflammation, toll-like receptor activation	+		
**SERPINB5**	30.1	Maspin, serine protease inhibitor with tumor suppressor properties			+
**CXCL10**	25.5	Immune-stimulating chemokine	+	+	
**CXCL13**	20.7	Immune-stimulating chemokine	+		
**CXCL9**	17.4	Immune-stimulating chemokine	+		
**LAMB3**	15	Laminin 5—target of Smad4 tumor suppressor			+
**LAMC2**	15	Laminin 5—target of Smad4 tumor suppressor			+
**S100A9**	14.8	Migration inhibitory factor-Inflammation, toll-like receptor activation	+		
**CXCL11**	10.9	Immune-stimulating chemokine	+	+	
**ITGB8**	10.8	Integrin αvβ8 T-cell homeostasis	+		
**CXCL8**	10.6	Immune-stimulating chemokine	+		
**IL1RN**	10.0	IL-1 receptor antagonist associated with suppression of early carcinogenic events in oral malignancies	+		+
**PLA2G2A**	−90.91	Phospholipase A2—inflammation, stroma, and cell cycle activities	+	+	+
**CCL18**	−27.03	Immunosuppressive chemokine	+	+	
**CCL14**	−25.64	Immunosuppressive chemokine—activates M2 monocytes	+	+	
**SFRP1**	−24.39	Wnt signaling, Th17	+	+	+
**CD209**	−22.22	DC-SIGN—immunosuppressive modulatory receptor—L10 induction	+		
**MARCO**	−18.18	Immunosuppressive tumor-associated macrophage (TAM) receptor	+		
**RELN**	−17.24	Reelin—upregulated in multiple tumor types; stroma formation; IL10 expression	+	+	
**NGFR**	−14.49	Nerve growth factor receptor—oncogenic inhibitor of p53			+
**GAS1**	−10.10	Pleiotropic cell cycle regulator; cooperativity with hedgehog			+
**PRLR**	−10.00	Prolactin receptor			+

Strongly modulated genes effected by p53 treatment with at least a tenfold change in expression represented a highly, statistically significant gene subset compared to genes with less than a tenfold change from baseline (*p* value < 0.00001 by two-sided Fisher’s Exact test). These genes were grouped into immune modulatory, stroma/fibrosis, and tumor suppressor/cell cycle functional categories.

With respect to immune response modulating genes, expression of the proinflammatory S100A8 and S100A9 genes were upregulated post-treatment by 35- and 15-fold, respectively. These genes are involved in pattern recognition receptor (PRR), damage-associated molecular patterns (DAMPs), and pathogen-associated molecular patterns (PAMPs), which are key to the initiation of immune responses. The IFN gamma-regulated chemokines CXCL8,9,10,11,13 were all upregulated by 10- > 25-fold, reflecting their role in antitumor immune responses. The gene encoding Serpin B5 (maspin) was upregulated by >30-fold and recent data indicate that maspin expression correlates with the activation and proliferation of CD8 + T-cell subsets and thus can modify the host immune response [19, 20].

In regard to the downregulation of gene expression contributing to increased antitumor immune responses, PLA2G2A which suppresses interferon-induced genes [21] had the greatest downregulation by >90-fold after Ad-p53 treatment. In addition, PLA2G2A is a direct target for beta-catenin-dependent Wnt signaling [22] and has been implicated in the regulation of Notch, TGF-beta, and Hedgehog signaling pathways [22]. The Wnt-beta-catenin and TGF-beta signaling pathways contribute to a lack of T-cell infiltration in tumors and inhibit immune checkpoint blockade therapy [23, 24]. The principal effector of the Wnt pathway, the CTNNB1 gene encoding beta-catenin was decreased by 3.6-fold, reflecting decreases in multiple components of beta-catenin signaling. The Ad-p53 therapy resulted in a decrease in the immune-suppressive chemokines CCL18 by >27-fold and CCL14 by >25-fold. CD209 (DC-SIGN), MARCO, and RELN genes function in the downregulation of the immune system through IL10 and inhibitory tumor-associated macrophage mechanisms respectively [25–27]. CD209 is downregulated by >20-fold, MARCO is downregulated by >18-fold, and RELN by >17-fold.

Regarding antistromal/fibrosis effects, several chemokine genes associated with stoma/fibrosis formation were downregulated by 9–27-fold, including CCL18, CXCL14, CXCL12. Another fibrosis-related gene is secreted frizzled receptor 1 (sFRP1), which was downregulated by >24-fold. Multiple genes with antistromal/fibrosis effects were upregulated by Ad-p53 treatment. CXCL10 and CXCL11 are known to attenuate bleomycin-induced pulmonary fibrosis CXCL10 and CXCL11 were increased by 25- and >10-fold, respectively, reflecting an antifibrotic activity of Ad-p53. Similarly, low levels of IL-1RN (IL-1 receptor antagonist) are associated with idiopathic pulmonary fibrosis and the IL-1RN gene was upregulated by tenfold.

The gene showing the greatest upregulation after the Ad-p53 treatment was the transcription factor SOX2 (42-fold upregulation post-treatment). SOX2 (SRY-Box Transcription Factor 2) is associated with repressing tumorigenic HPV transcription [28]. The gene encoding Serpin B5 (maspin) was upregulated by >30-fold and has tumor suppressor antiangiogenic functions. Other highly upregulated genes with tumor suppressor and/or cell cycle inhibitory activities are LAMB3, LAMC2, and IL1RN, which were increased by 10- to 15-fold. PLA2G2A and SFRP1, which are associated with oncogenic cell cycling activity were downregulated by 90- and 24-fold, respectively. Similarly, NGFR, GAS1, and PRLP have pleiotropic cell cycling properties and were inhibited by 14.49- to 10-fold following the Ad-p53 treatment.

In addition to the individual genes in the Nanostring IO 360 dataset, the pre- and post-treatment biopsies data were also analyzed for gene signatures associated with immune activation, immune suppression, and antistromal/fibrosis functions. As shown in Fig. 8, Interferon-gamma, CD8 + T-cell profiles, Cytotoxicity and iNOS (inducible nitric oxide synthase, NOS2) profiles were increased consistent with activation of antitumor immune responses, whereas immunosuppressive pathways exemplified by IL-10 and TGF-beta and stroma signatures were downregulated, respectively.

Surprisingly, in addition to modulating immune mediators for antitumor immune responses, the Ad-p53 therapy downregulated multiple gene pathways implicated in stroma/fibrosis formation. The stroma-related gene pathway (which comprises >50 gene products (see Supplemental Table 1) encompassing extracellular matrix remodeling, cell adhesion, myeloid cells, collagens, angiogenesis, and metastasis was unexpectedly, strongly downregulated by Ad-p53 treatment.

## Discussion

While immune checkpoint inhibitors are being increasingly employed in cancer treatment, most cancer patients do not respond to this form of immunotherapy [1]. Similarly, genetically engineered versions of IL2 and IL15 cytokines with selective CD122/132 receptor activation have reduced toxicities and greater efficacy than their native proteins but the majority of tumors do not respond to these treatments either [2, 3]. The syngeneic B16F10 melanoma is known to be resistant to these immunotherapies and is a useful model to explore novel immunotherapeutic approaches. In our studies, loco-regional Ad-p53 tumor suppressor gene therapy reversed resistance to both immune checkpoint inhibitor and selective CD122/CD132 IL2 and IL15 therapies, demonstrating unexpected synergies with abscopal effects on distant tumors that were not treated with Ad-p53. Remarkably, the “Triplet” therapy combining Ad-p53 with selective CD122/CD132 agonists and immune checkpoint blockade resulted in the complete tumor remissions and potentially curative outcomes that significantly surpassed the efficacy of all other doublet and monotherapies tested, which did not generate complete responses nor extended survivals.

These promising preclinical results led to the initiation of a Phase 1/2 clinical trial of combined Ad-p53 and anti-PD-1 therapy in patients with recurrent HNSCC. As reported elsewhere [29], preliminary evaluation of pre- and post-Ad-p53 treatment biopsies were evaluated for changes in gene-expression profiles and revealed increased interferon signaling, CD8 + T-cell signaling and the tumor inflammation signature, which are all associated with increased responses to immune checkpoint inhibitors [17, 18, 30]. The Ad-p53 treatment also decreased known immune-suppressive TGF-beta and beta-catenin signaling, which may also contribute to enhanced immune checkpoint inhibitor efficacy.

In the present report, we examined the gene signatures associated with the Ad-p53 treatment more thoroughly to provide additional insights into the potential mechanism of actions for the observed synergies with IL2/IL15 agonists and immune checkpoint blockade. We identified 23 strongly modulated genes with at least a tenfold change in expression representing a highly, statistically significant gene subset most substantially effected by Ad-p53 treatment. These genes may be grouped into immune modulatory, stroma/fibrosis, and tumor suppressor/cell cycle functional categories. Unexpectedly, many of these genes were found to be involved in immune responses and antistroma/fibrosis functions, which are not typically associated with p53 tumor suppressor mechanisms of action.

With respect to immune response modulating genes, expression of the proinflammatory S100A8 and S100A9 genes were up-regulated post-treatment by 35- and 15-fold, respectively. These genes are involved in pattern recognition receptor (PRR), damage-associated molecular patterns (DAMPs), and pathogen-associated molecular patterns (PAMPs), which are key to the initiation of immune responses [31]. The IFN gamma-regulated chemokines CXCL8,9,10,11,13 were all upregulated by 10- > 25-fold, reflecting their role in antitumor immune responses. The gene encoding Serpin B5 (maspin) was upregulated by >30-fold and data indicate that the maspin expression correlates with the activation and proliferation of CD8 + T-cell subsets and thus can modify the host immune response [19, 20]. Additional mechanistic studies, particularly involving an analysis of the cell types mediating antitumor immune responses will be needed to confirm and extend these initial gene signature findings.

In regard to the downregulation of gene expression contributing to increased antitumor immune responses, PLA2G2A which suppresses interferon-induced genes [21] had the greatest downregulation by >90-fold after the Ad-p53 treatment. In addition, PLA2G2A is a direct target for beta-catenin-dependent Wnt signaling [22] and has been implicated in the regulation of Notch, TGF-beta, and Hedgehog signaling pathways [21]. The Wnt-beta-catenin and TGF-beta signaling pathways contribute to a lack of T-cell infiltration in tumors and inhibit immune checkpoint blockade therapy [23, 24]. The principal effector of the Wnt pathway, the CTNNB1 gene encoding beta-catenin was decreased by 3.6-fold, reflecting decreases in multiple components of beta-catenin signaling. The Ad-p53 therapy resulted in a decrease in the immune-suppressive chemokines CCL18 by >27-fold and CCL14 by >25-fold. CD209 (DC-SIGN), MARCO, and RELN genes function in the downregulation of the immune system through IL10 and inhibitory tumor-associated macrophage mechanisms, respectively [25–27]. CD209 is downregulated by >20-fold, MARCO is downregulated by >18-fold, and RELN by >17-fold.

Surprisingly, in addition to modulating immune mediators for antitumor immune responses, Ad-p53 therapy downregulated multiple gene pathways implicated in stroma/fibrosis formation. The stroma-related gene pathway (which comprises >50 gene products (see Supplemental Table 1) encompassing extracellular matrix remodeling, cell adhesion, myeloid cells, collagens, angiogenesis, and metastasis was unexpectedly, strongly downregulated by Ad-p53 treatment. Several chemokine genes associated with stoma/fibrosis formation [32] were downregulated by 9- to 27-fold, including CCL18, CXCL14, CXCL12. Another fibrosis-related gene is secreted frizzled receptor 1 (sFRP1) [33], which was downregulated by >24-fold. Multiple genes with antistromal/fibrosis effects were upregulated by Ad-p53 treatment. CXCL10 and CXCL11 are known to attenuate bleomycin-induced pulmonary fibrosis [34] and were increased by 25- and >10-fold, respectively, reflecting antifibrotic activity of Ad-p53. Similarly, low levels of IL-1RN (IL-1 receptor antagonist) are associated with idiopathic pulmonary fibrosis [35] and the IL-1RN gene was upregulated by tenfold consistent with antifibrosis effects.

Regarding tumor suppressor/cell cycle inhibitory functions, the gene showing the greatest upregulation after Ad-p53 treatment was the transcription factor SOX2 (42-fold upregulation post-treatment). SOX2 (SRY-Box Transcription Factor 2) is associated with the repression of tumorigenic HPV transcription [28]. The gene encoding Serpin B5 (maspin) was upregulated by >30-fold and has tumor suppressor and antiangiogenic functions [36]. Other highly upregulated genes with tumor suppressor and/or cell cycle inhibitory activities are laminin-5 (LAMB3, LAMC2) [37] and IL1RN, which were increased by 10- to 15-fold. PLA2G2A [21] and SFRP1 [33] which are associated with oncogenic cell cycling activity were downregulated by 90- and 24-fold, respectively. Similarly, NGFR [38], GAS1 [39], and PRLP [40] have pleiotropic cell cycling properties and were inhibited by 14.49- to 10-fold following Ad-p53 treatment. Of particular relevance, NGFR is known to inhibit p53 and NGFR ablation enhances p53 activity [40].

In summary, loco-regional Ad-p53 tumor suppressor gene therapy reversed resistance to both immune checkpoint inhibitor and selective CD122/CD132 IL2 and IL15 therapies with substantial synergies. Remarkably, the “Triplet” therapy combining Ad-p53 with selective CD122/CD132 agonists and immune checkpoint blockade resulted in the complete tumor remissions and potentially curative outcomes that significantly surpassed the efficacy of all other doublet and monotherapies tested none of which resulted in complete responses nor extended survivals. With respect to potential mechanisms of action, gene-expression profiling comparing pre- and post-Ad-p53 tumor biopsies showed strong upregulation of genetic pathways involved in antitumor immune responses, including IFN-gamma activation, an increased CD8 + T-cell signature, with concomitant downregulation of TGF-beta and IL10 gene profiles. Unexpectedly, the Ad-p53 treatment substantially reduced fibrotic/stroma gene pathways. A number of previously unidentified, strongly p53 downregulated genes associated with stromal pathways and IL10 expression identified novel anticancer therapeutic applications. Ad-p53 treatment also decreased immune-suppressive TGF-beta and beta-catenin signaling, which may also contribute to enhanced immune checkpoint inhibitor efficacy. These mechanistic gene profiling insights should be confirmed in a larger number of treatment samples and combined with analyses of the cell types mediating antitumor immune responses to extend our initial findings. The evaluation of the safety of the combined treatments will also need to be assessed in future development studies. Taken together, our initial results imply the ability of Ad-p53 to induce efficacious local and systemic antitumor immune responses with the potential to reverse resistance to immune checkpoint inhibitor therapy when combined with IL2 and IL15 CD122/132 agonists supporting further clinical development of this triplet therapy.

## Supplementary information

Supplemental Figure 1.

Supplemental Figure 2.

Supplemental Table 1.

## Data Availability

The data supporting the conclusions of this article are either incorporated in the manuscript, its supplemental section or available by the corresponding author upon reasonable request.
