# Supplementary material for: Tumor suppressor immune gene therapy to reverse immunotherapy resistance
Source: Cancer Gene Ther. 2021 Aug 5;29(6):825–34. doi: 10.1038/s41417-021-00369-7 (PMC9209327; doi:10.1038/s41417-021-00369-7)
Supplement: Supplementary file 1 — Supplemental Figure 1. [file 41417_2021_369_MOESM1_ESM.docx]

**Supplemental Figure 1**


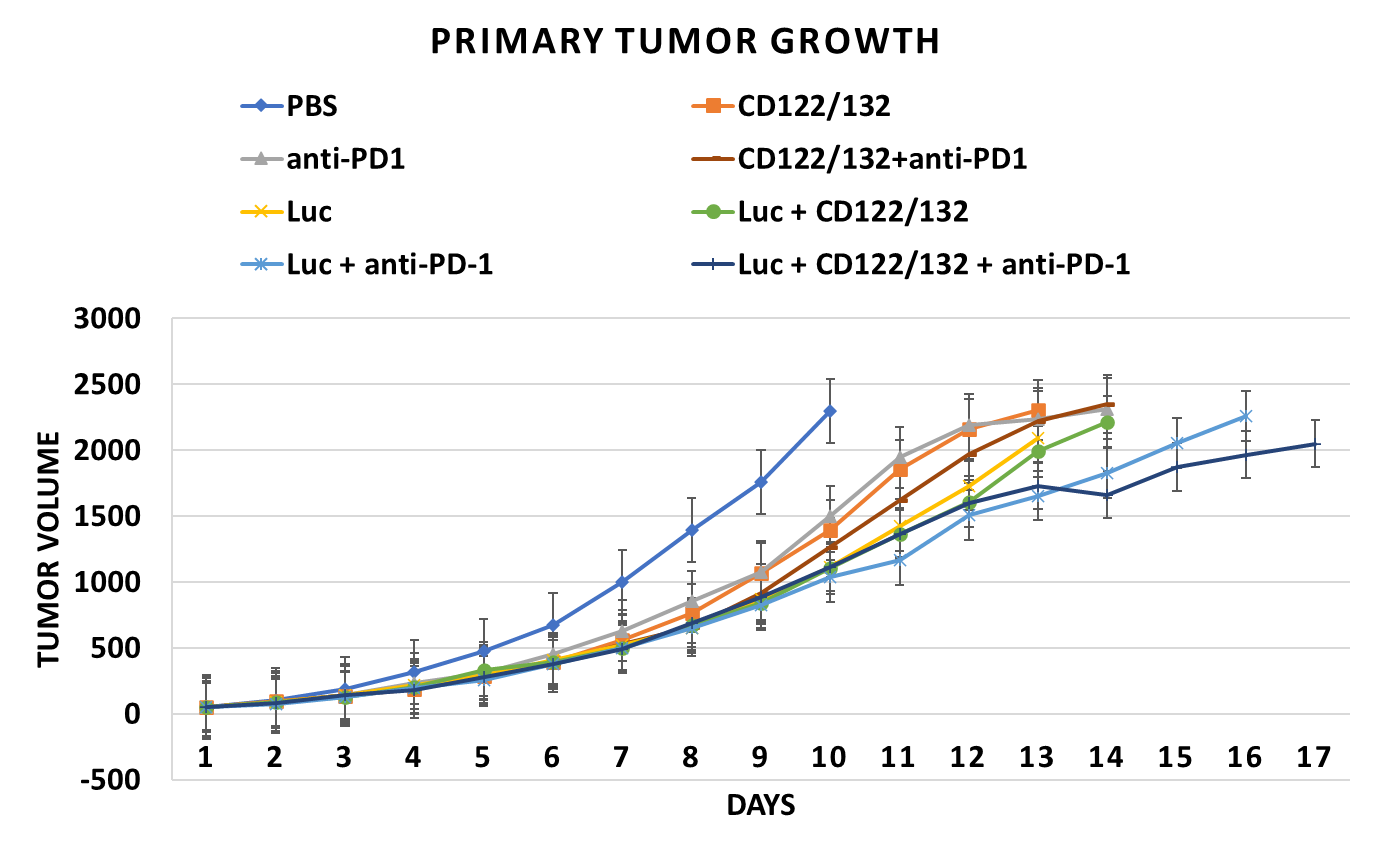


***Supplemental Figure 1. Ad-Luciferase (Ad-Luc) negative control + IL2 CD122/132 agonist + anti-PD-1 Efficacy: Tumor Volume.*** *A graph showing primary tumor volumes over time in mice receiving either phosphate buffered saline (PBS) control, CD122/132, anti-PD-1, IL2 CD122/132 + anti-PD-1, Ad-Luc control, or the combination of Ad-Luc control + IL2 CD122/132, Ad-Luc control + anti-PD-1 and Ad-Luc control + IL2 CD122/132 + anti-PD-1. In contrast to the treatments with Ad-p53, VirRx007 and Ad-IL24, there was no significant increase in therapeutic efficacy when Ad-Luc was combined with anti-PD-1, IL2 CD122/132, or IL2 CD122/132 + anti-PD-1 treatments. By day 16, the mean tumor volumes for all groups exceeded 2,000 mm^3^. A statistical analysis of variance (ANOVA) comparison of tumor volumes on Day 16 was not statistically significant (p-value = 0.1212; none of the mean tumor volumes between any of the treatment groups were statistically significant). Tumor Volume = mm^3^.*
