# Supplementary material for: Tumor suppressor immune gene therapy to reverse immunotherapy resistance
Source: Cancer Gene Ther. 2021 Aug 5;29(6):825–34. doi: 10.1038/s41417-021-00369-7 (PMC9209327; doi:10.1038/s41417-021-00369-7)
Supplement: Supplementary file 2 — Supplemental Figure 2. [file 41417_2021_369_MOESM2_ESM.docx]

**Supplemental Figure 2**


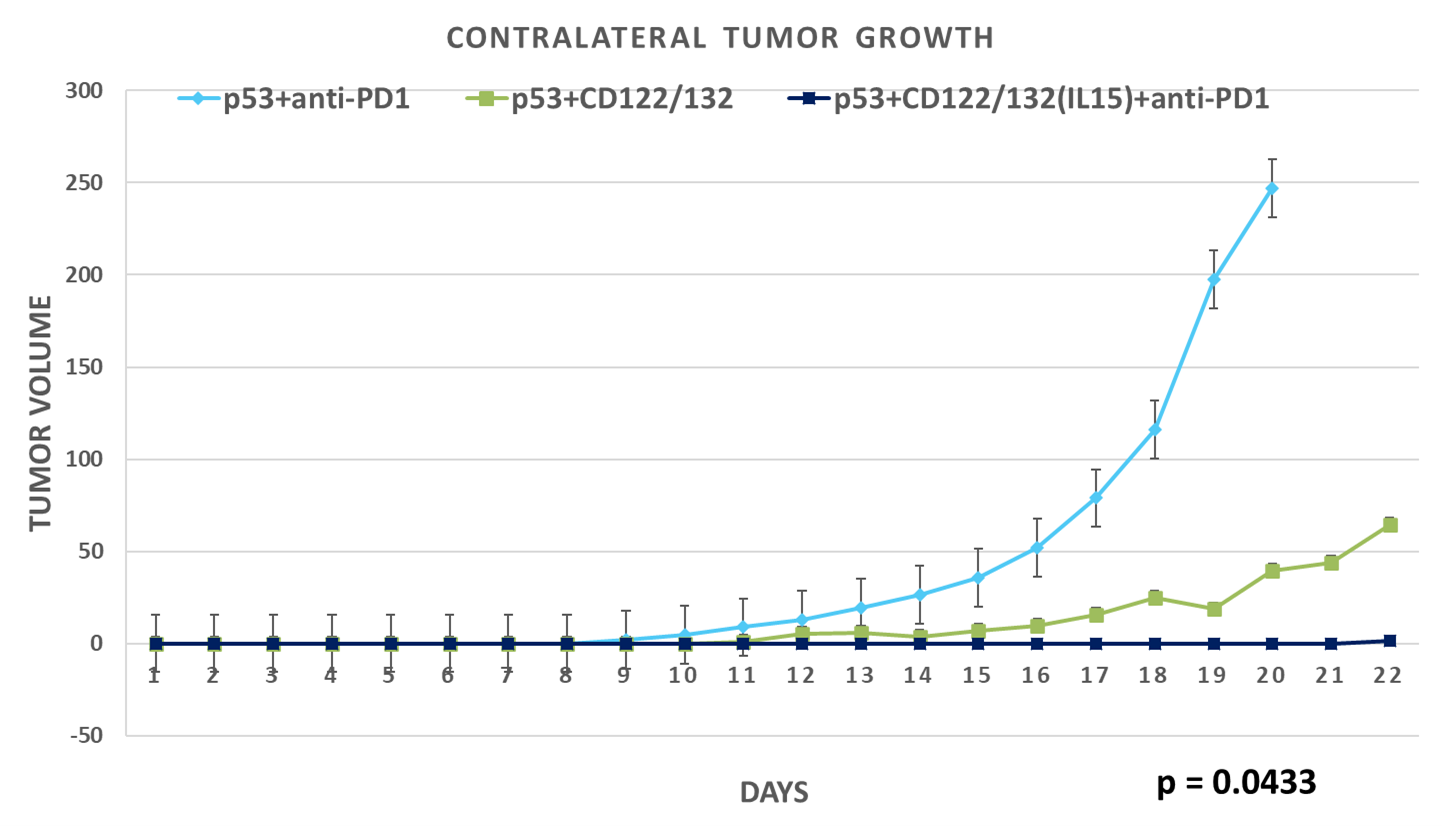


***Supplemental Figure 2. Substantially Superior Abscopal/Systemic Efficacy of “Triplet” Ad-p53 + IL15 CD122/132 agonist + anti-PD-1 Therapy.*** *In this experiment, the preferential CD122/CD132 agonist was an immunocomplex comprised of recombinant IL15 and IL-15-R alpha-Fc. Consistent with the unexpected, substantially increased synergistic effects of Ad-p53 + IL15 CD122/132 + anti-PD-1 treatment on primary tumor growth, we also observed a surprisingly powerful and statistically significant abscopal effect of triplet Ad-p53 + IL15 CD122/132 + anti-PD-1 treatment compared to the other Ad-p53 treatment groups. A statistical analysis of variance (ANOVA) comparison of these contralateral tumor volumes determined synergy of the anti-tumor effects of Ad-p53 + IL15 CD122/132 + anti-PD-1 treatment (p-value = 0.0433 overall). Only the Ad-p53 + CD122/132 + anti-PD-1 group demonstrated a statistically significant decrease in contralateral tumor growth vs. the Ad-p53 + anti-PD-1 group (*p-value = 0.0359*). Tumor Volume = mm^3^.*
