# Supplementary material for: Tumor suppressor immune gene therapy to reverse immunotherapy resistance
Source: Cancer Gene Ther. 2021 Aug 5;29(6):825–34. doi: 10.1038/s41417-021-00369-7 (PMC9209327; doi:10.1038/s41417-021-00369-7)
Supplement: Supplementary file 3 — Supplemental Table 1. [file 41417_2021_369_MOESM3_ESM.docx]

**Supplemental Table 1. Stromal-related gene signature from IO360 panel**

| **Stromal Factor Gene Signature** | | | | | |
| --- | --- | --- | --- | --- | --- |
| A2M | COL4A5 | ITGA4 | KDR | NID2 | TGFB2 |
| BMP2 | COL5A1 | ITGA6 | LAMA1 | PDGFA | TGFB3 |
| CASP3 | COL6A3 | ITGAE | LAMB3 | PDGFB | THBS1 |
| CD36 | COMP | ITGAL | LAMC2 | PECAM1 | VCAM1 |
| CD44 | CTSS | ITGAM | LOXL2 | PLOD2 | VCAN |
| CD47 | ICAM1 | ITGAV | LTBP1 | PRKCA |  |
| CDH1 | ICAM2 | ITGAX | MMP1 | RELN |  |
| COL11A1 | ICAM3 | ITGB2 | MMP7 | SERPINH1 |  |
| COL11A2 | ITGA1 | ITGB3 | MMP9 | SPP1 |  |
| COL17A1 | ITGA2 | ITGB8 | NCAM1 | TGFB1 |  |
